# DELTA-SoyStage: A Lightweight Detection Architecture for Full-Cycle Soybean Growth Stage Monitoring †

**DOI:** 10.3390/s25237303

**Published:** 2025-12-01

**Authors:** Abdellah Lakhssassi, Yasser Salhi, Naoufal Lakhssassi, Khalid Meksem, Khaled Ahmed

**Affiliations:** 1 School of Computing, Southern Illinois University, Carbondale, IL 62901, USA; abdellah.lakhssassi@siu.edu; 2 Department of Plant, Soil, and Agricultural Systems, Southern Illinois University, Carbondale, IL 62901, USA; yasser.salhi@siu.edu (Y.S.); meksem@siu.edu (K.M.); 3 Department of Biological Sciences, School of Science, Hampton University, Hampton, VA 23668, USA; naoufal.lakhssassi@hamptonu.edu

**Keywords:** soybean phenology, growth stage detection, transformer-based models, crop monitoring, computer vision, object detection, RGB image

## Abstract

The accurate identification of soybean growth stages is critical for optimizing agricultural interventions, where mistimed treatments can result in yield losses ranging from 2.5% to 40%. Existing deep learning approaches remain limited in scope, targeting isolated developmental phases rather than providing comprehensive phenological coverage. This paper presents a novel object detection architecture DELTA-SoyStage, combining an EfficientNet backbone with a lightweight ChannelMapper neck and a newly proposed DELTA (Denoising Enhanced Lightweight Task Alignment) detection head for soybean growth stage classification. We introduce a dataset of 17,204 labeled RGB images spanning nine growth stages from emergence (VE) through full maturity (R8), collected under controlled greenhouse conditions with diverse imaging angles and lighting variations. DELTA-SoyStage achieves 73.9% average precision with only 24.4 GFLOPs computational cost, demonstrating 4.2× fewer FLOPs than the best-performing baseline (DINO-Swin: 74.7% AP, 102.5 GFLOPs) with only 0.8% accuracy difference. The lightweight DELTA head combined with the efficient ChannelMapper neck requires only 8.3 M parameters—a 43.5% reduction compared to standard architectures—while maintaining competitive accuracy. Extensive ablation studies validate key design choices including task alignment mechanisms, multi-scale feature extraction strategies, and encoder–decoder depth configurations. The proposed model’s computational efficiency makes it suitable for deployment on resource-constrained edge devices in precision agriculture applications, enabling timely decision-making without reliance on cloud infrastructure.

## 1. Introduction

Soybeans (Glycine max) represent one of the world’s most economically significant crops, serving as a primary source of protein and oil for global food and feed systems. Soybean oil accounts for over 25% of global vegetable oil production, ranking as the second-largest vegetable oil globally after palm oil [1]. The crop’s global importance stems from its unique nutritional composition, containing 40–41% protein and 8.1% to 24.0% oil content, making it particularly valuable for both human consumption and animal feed applications [2]. Soybeans are processed into numerous products across food, feed, and industrial sectors, with traditional and modern applications ranging from industrial applications to functional foods with cardiovascular health benefits [3]. The major producing countries—the United States, Brazil, and Argentina—collectively account for approximately 82% of global soybean production, with approximately 98% of globally produced soybean meal utilized for animal nutrition [4].

In the United States, soybeans represent the second-largest row crop by area and economic value, with significant agricultural and economic impact. The U.S. Department of Agriculture (2024) reports that in 2023, 33.8 million hectares of soybeans were planted, producing 4.16 billion bushels with a total crop value exceeding USD 60.7 billion, with projections for record-high production of 4.6 billion bushels in 2024/25 [5,6,7]. More than 80% of U.S. soybeans are grown in the northern Midwest region, with Iowa, Illinois, Minnesota, and Indiana as the top four producing states—collectively accounting for over 49% of the annual U.S. supply [4]. The economic significance is further demonstrated by the 2023 production of 48 million metric tons of soybean meal valued at USD 498.14 per metric ton and 11.9 million metric tons of soybean oil at USD 1,439 per metric ton [4].

Soybean growth is divided into two main stages: vegetative (V) and reproductive (R) [8]. The vegetative stage covers early plant development (VE - VN), starting from seedling emergence (VE) through the formation of successive nodes and leaves. In the VE (emergence) stage, soybean development begins by breaking through the soil (Germinating) under three adequate conditions: soil moisture, temperature, and oxygen [8]. By VC (cotyledon stage), the first unifoliolate leaves are fully open, marking the start of true leaf growth. At each subsequent stage, V1, V2, V3, VN (N number of nodes on the main stem), and so on, correspond to the number of fully expanded and unrolled trifoliolate leaves on the main stem. This continues until the plant reaches its final vegetative stage.

The reproductive stage starts at R1 (beginning bloom) when the first flower appears on the main stem, followed by R2 (full bloom) when the flowers are visible at the upper nodes. The plant then transitions to R3 (beginning pod) with pods of 3/16 inch long, and R4 (full pod), where small green pods of 3/4 inch long form and grow to full size [8]. At R5 (beginning seed), seeds of 1/8 inch long start to develop inside the pods, and by R6 (full seed), they fill the pod cavity, where the pods appear plump with fully filled green seeds [8]. When the plant reaches R7 (beginning maturity), at least one pod turns yellow-brown, signaling the start of drying and physiological maturity. The leaves may start yellowing and dropping at this stage. Finally, at R8 (full maturity), nearly all pods have reached their mature color, and the plant is ready for harvest. Usually, 5 to 10 days of good drying weather after R8 are needed before harvest, when seeds have less than 15% moisture.

The accurate identification of soybean growth stages is critical for optimizing pesticide and herbicide applications, as mistimed treatments can result in yield losses ranging from 2.5% to 40% and economic penalties of USD 5–80 per acre, depending on weed density and timing [9]. Research demonstrates that herbicides must be applied at V3–V4 stages to achieve effective weed control, with delays beyond V4 causing exponential yield losses—particularly under high weed pressure, where applications at V4 already result in up to 25% yield loss [9]. For insecticides, the optimal windows are equally narrow: soybean aphid control is most effective when applied at the R2–R3 growth stages once populations reach the economic threshold of 250 aphids per plant [10], while stink bug management requires applications during the reproductive growth stages—when adults and nymphs colonize pods and developing seeds—which are key to preventing pod loss, seed deformation, and yield decline [11]. These precise intervention windows underscore that growth stage identification is not merely a best practice but an essential skill for profitable and sustainable soybean production.

Deep learning has emerged as a transformative tool in agricultural phenotyping, particularly for recognizing plant growth stages. Convolutional Neural Networks (CNNs) have shown strong performance when applied to RGB images captured by UAVs, smartphones, and stationary cameras. These models can learn stage-specific visual cues—such as changes in leaf texture, shape, and canopy structure—and have been used to detect and classify growth stages ranging from seedling emergence to reproductive development. As the cost of aerial imaging and computing continues to decrease, the deployment of such models in real-world settings has become increasingly feasible across multiple crops.

The advancement of affordable imaging sensors has played a key role in enabling automated phenological monitoring and transforming smart agriculture globally [12,13]. Modern RGB camera sensors, including those integrated in smartphones and UAV-mounted cameras, provide high-resolution imagery suitable for growth stage detection at costs that are orders of magnitude lower than specialized agricultural sensors [14]. Recent AI-powered aerial imaging systems utilizing these RGB sensors have achieved over 90% accuracy in identifying crop diseases and developmental stages [15]. When combined with edge computing platforms equipped with GPUs and AI processing capabilities, these imaging sensors enable distributed monitoring networks that can operate autonomously in field conditions, supporting precision monitoring, early crop health diagnostics, yield forecasting, and data-driven decision support [13].

The integration of RGB imaging sensors with complementary sensor modalities represents a transformative direction for comprehensive crop monitoring systems. Hyperspectral sensors, capturing hundreds of narrow spectral bands beyond visible light, enable AI models to detect subtle biochemical stress indicators such as water stress or nitrogen deficiencies that RGB imagery cannot discern, greatly improving crop health assessments and yield prediction accuracy [15,16]. Thermal imaging sensors provide canopy temperature measurements for water stress monitoring and irrigation optimization [17,18]. Networks of environmental IoT sensors measuring soil moisture, temperature, and humidity feed real-time data to AI platforms that predict optimal irrigation timing, forecast yields, and provide decision support for fertilizer and pest control measures [19,20,21]. By fusing multi-source data from optical cameras, spectral sensors, and IoT devices, modern AI-driven farming systems deliver real-time, site-specific insights and recommendations, enabling early interventions that improve productivity and resource-use efficiency while promoting sustainability [15,21].

Object detection models based on CNNs have been used to identify growth stages in field and greenhouse settings across various crops and weeds [22,23]. An enhanced YOLOv4 model integrating DenseNet achieved high accuracy and speed in detecting different developmental stages of fruit in orchard environments, reaching a mean average precision (mAP) of 96.2% and F1-score of 93.6% at 44.2 FPS [24]. UAV imagery of weed phenology was also processed using a suite of object detectors, where lightweight YOLOv5 models offered real-time performance, and RetinaNet attained an AP of 87.5% for stage-specific weed presence [22]. In rice fields, an improved YOLOv8 model with a MobileNetv3 backbone and coordinate attention modules achieved a mAP of 84% on high-resolution UAV images [25]. For horticultural crops such as strawberries, a YOLOX-based lightweight detector demonstrated near-perfect performance in identifying ripening stages using greenhouse images [26]. These results confirm that state-of-the-art CNN detectors can localize and classify plant development stages with high accuracy and, in many cases, real-time speed.

Beyond object detection, CNN-based classifiers and segmentation models have been used for pixel-level labeling and sequence-based stage prediction. High-performing architectures such as VGG19 and DenseNet have been employed for general crop growth classification from field and time-lapse imagery, yielding consistently high accuracy [27,28]. A hybrid model combining ResNet34 with attention mechanisms was developed for maize and achieved over 98% classification accuracy on UAV orthophotos, further deployed as a web-based tool for field use [29]. For finer pixel-level recognition, a U-Net-based segmentation model was enhanced to identify maize stages in the field, achieving a mean Intersection over Union (IoU) of 94.51% and pixel-level accuracy of 96.93% [30]. Temporal models have also been integrated with CNNs; for instance, a ResNet50 + LSTM fusion model was used to classify wheat growth stages from time-series camera data, reaching 93.5% accuracy [31]. These hybrid models illustrate the potential of combining spatial and temporal information for phenological classification.

Modern CNN-based models have been applied to soybean growth stage recognition, with a focus on optimizing both inference speed and accuracy. Lightweight variants of YOLOv5 have been trained to detect flowering and reproductive stages, often using UAV imagery with real-time performance targets. Pruned YOLOv5 architectures and attention-augmented backbones have demonstrated strong results for both in-field and edge-deployed phenology applications. In another line of research, over 44,000 strawberry images were used to train CNN classifiers for fine-grained seven-stage classification, achieving over 83% test accuracy with EfficientNet and similar models [32]. These applications demonstrate the feasibility of deploying accurate and efficient CNNs across diverse environments, including field, greenhouse, and mobile platforms.

While recent advances in deep learning have improved the detection of certain soybean growth stages, most studies remain limited in scope, often targeting isolated phases such as early vegetative development. For example, early-stage detection efforts have utilized classical CNN architectures like AlexNet and its improved variants to classify VE (emergence), VC (cotyledon), and V1 (first trifoliolate) stages [33]. However, these approaches do not leverage the capabilities of more recent and efficient architectures, such as transformer-based models or hybrid attention-enhanced CNNs, which offer greater accuracy and robustness in real-world applications.

In parallel, other research has focused on broader agronomic outcomes, including yield prediction based on pod detection [34] or overall maturity estimation using aerial imagery and conventional machine learning classifiers such as random forests [35]. While valuable, these studies do not provide stage-specific granularity across the full phenological timeline. As a result, there remains a significant gap in the literature for models that can comprehensively detect and classify all key soybean growth stages—from emergence and vegetative development to flowering, podding, and maturation—under varying environmental and imaging conditions. Addressing this gap is essential for advancing phenological monitoring, precision agriculture, and decision support systems in soybean production.

In prior work, a transformer-based model—SoyStageNet—was developed for the real-time classification of six early soybean growth stages (VE to VN) using RGB imagery captured in greenhouse conditions [36]. While this approach demonstrated strong accuracy and efficiency in early-stage detection, it was limited in scope and did not address later developmental phases such as flowering, podding, and maturity. The present study extends this line of research by expanding the stage coverage to nine classes representing the full phenological cycle, and by designing a new lightweight architecture aimed at achieving state-of-the-art accuracy while preserving real-time inference performance in practical deployment scenarios.

The main contributions of this work are as follows:

- We present a comprehensive dataset of 17,204 labeled RGB images spanning nine complete soybean growth stages from emergence (VE) through full maturity (R8), addressing the limitation of existing datasets that focus only on isolated developmental phases.
- We introduce DELTA-SoyStage, a novel object detection architecture combining an EfficientNet backbone with a lightweight ChannelMapper neck and the newly proposed DELTA (Denoising Enhanced Lightweight Task Alignment) detection head, achieving 73.9% AP with only 24.4 GFLOPs—4.2× fewer FLOPs than state-of-the-art baselines while maintaining competitive accuracy.
- We conduct extensive ablation studies systematically evaluating task alignment mechanisms, multi-scale feature extraction strategies, and encoder–decoder depth configurations, providing insights into optimal architectural choices for agricultural detection applications.
- Through computational analysis, we show that our architecture’s reduced FLOP requirements and compact parameter count position it for deployment on resource-constrained edge devices, potentially enabling timely agricultural decision-making without reliance on cloud infrastructure.

The rest of this paper is organized as follows: Section 2 describes our comprehensive soybean growth stage dataset collection methodology and annotation process. Section 3 presents the proposed DELTA-SoyStage architecture, detailing the EfficientNet backbone, lightweight ChannelMapper neck, and novel DELTA detection head. Section 4 provides implementation details and evaluation metrics. Section 5 presents quantitative comparisons with state-of-the-art methods, extensive ablation studies validating our design choices, and qualitative analysis of detection results. Finally, Section 6 concludes the paper with a discussion of limitations and future research directions.

## 2. Dataset

A total of 160 soybean seeds, comprising 20 seeds from each of eight different wild-type species, were planted in a controlled greenhouse environment. The seeds were specifically selected for their diverse phenotypic characteristics, including flower color, plant height, leaf shape and pod color. The greenhouse was maintained at a constant temperature of 27 °C to ensure optimal growth and germination conditions.

At the VC stage (cotyledon stage), seedlings were transplanted into individual pots. Irrigation was conducted every two to five days, adjusting frequency based on the plants’ growth phase. Slow-release fertilizers were applied at the R1 stage (beginning bloom) to promote healthy development. Simultaneously, image acquisition was performed using the RGB camera sensor of a Samsung Galaxy S20FE smartphone (Samsung Electronics, Suwon, Republic of Korea). Photos were taken from multiple angles in an orbiting pattern from the top, ensuring varied lighting conditions, backgrounds, and plant orientations to create a diverse and representative dataset.

Among the various species, PI567516C displayed notably accelerated growth, reaching late reproductive stages earlier than other accessions. During critical reproductive stages, irrigation frequency was increased to promote healthy pod development and seed filling. Once plants reached the R6 stage (full seed), watering was discontinued to prevent excessive moisture and facilitate proper seed drying and maturation.

**Figure 1:**
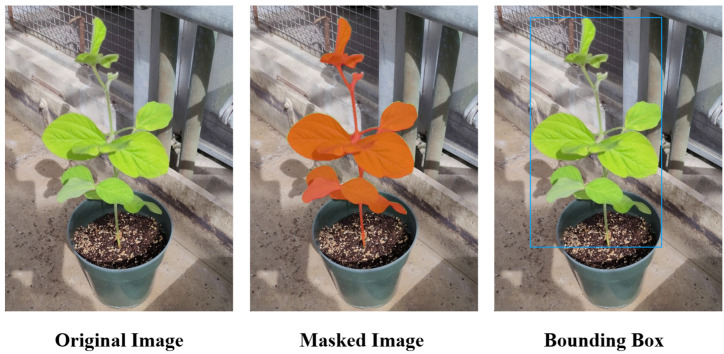
Annotation pipeline showing (**left**) the original image, (**center**) SAM2-based segmentation, and (**right**) manually refined bounding boxes.

To ensure accurate and efficient annotations, we initially employed SAM2 (Segment Anything Model 2) [37] for plant segmentation. However, SAM2 encountered challenges with precise segmentation due to varying backgrounds, inconsistent lighting conditions, and light reflections on leaf surfaces. Following initial segmentation, manual inspection and classification were conducted using LabelImg [38] to ensure precise bounding boxes and correct class assignments for all images (Figure 1).

Twenty-five soybean seeds from each of eight different wild-type species (200 plants total) were planted in a controlled greenhouse environment. Plants were monitored throughout their growth cycle, with video frames extracted every 40 frames to ensure temporal diversity and prevent data leakage from consecutive frames. The final dataset comprised 17,204 labeled images, with class distribution detailed in Table 1. We used an 80/10/10 split—13,763 images for training, 1720 for validation, and 1721 for testing—ensuring a well-balanced learning process for effective generalization across different growth stages and background conditions.

## 3. Methods

### 3.1. Preliminaries

Deep learning architectures for object detection are typically structured into three main components: the backbone, neck, and head [39]. This modular design enables independent optimization of feature extraction, feature aggregation, and prediction tasks, leading to more efficient and accurate models. The backbone serves as the primary feature extractor, processing input images to capture hierarchical features at various levels of abstraction. Modern backbones like ResNet introduced residual connections to mitigate vanishing gradient problems, enabling effective training of deeper networks [40]. Similarly, vision transformers such as Swin Transformer apply hierarchical attention mechanisms within local windows, efficiently capturing both local and global context for dense prediction tasks [41]. The neck aggregates and refines features from multiple backbone layers, typically employing Feature Pyramid Networks (FPN) to enhance multi-scale feature fusion [42]. This component is crucial for detecting objects across different scales by combining semantic information from various resolution levels. The head makes final predictions for object classification and bounding box regression using the aggregated features from the neck. Depending on the architecture, heads can be designed for region proposal generation (e.g., Faster R-CNN [43]) or direct detection (e.g., single-stage detectors like YOLO [44]).

Benchmark Models: To evaluate our proposed DELTA-SoyStage model, we benchmark against three state-of-the-art detectors representing different architectural paradigms: (1) DINO [45]—a transformer-based detector with contrastive denoising training that improves convergence and robustness; (2) TOOD [46]—a task-aligned one-stage detector addressing classification–localization misalignment through joint optimization; and (3) Faster R-CNN [43]—a two-stage detector integrating Region Proposal Networks for accurate region-based detection. Experimental Setup: Each of the three benchmark models is trained and evaluated with three different backbone architectures to ensure comprehensive comparison: ResNet-50 (R50) and ResNet-101 (R101) representing CNN-based feature extractors [40], and Swin Transformer representing vision transformer-based backbones [41]. This systematic evaluation across 9 model-backbone combinations (3 models × 3 backbones) provides robust baseline performance metrics against which our DELTA-SoyStage is compared.

### 3.2. EfficientNet Backbone

We adopt EfficientNet [47] as our backbone architecture, a highly efficient Convolutional Neural Network that systematically scales network dimensions through compound scaling to achieve superior accuracy–efficiency trade-offs.

EfficientNet is designed to optimize computational efficiency while maximizing feature extraction capability through a principled compound scaling approach that uniformly scales network depth, width, and input resolution. The backbone consists of four key components that work synergistically to provide robust multi-scale feature representations: (1) compound scaling methodology for balanced network scaling across all dimensions, (2) Mobile Inverted Bottleneck Convolution (MBConv) blocks for efficient feature extraction, (3) Squeeze-and-Excitation (SE) attention mechanisms for adaptive channel-wise feature recalibration, and (4) progressive feature extraction across multiple scales for hierarchical representation learning.

The compound scaling methodology represents a paradigm shift from conventional scaling approaches by simultaneously optimizing network depth (*d*), width (*w*), and input resolution (*r*) through a unified scaling coefficient ϕ:

(1) $$\begin{array}{cc} d & =\alpha^{\phi },\;w=\beta^{\phi },\;r=\gamma^{\phi }, \end{array}$$

(2) $$\begin{array}{cc} \mathrm{subject}\;\mathrm{to}:\; & \alpha \cdot \beta^{2}\cdot \gamma^{2}\approx 2,\;\alpha \geq 1,\beta \geq 1,\gamma \geq 1, \end{array}$$

where α, β, and γ are scaling coefficients determined through grid search, and the constraint ensures that computational cost (FLOPs) increases by approximately $2^{\phi }$.

The core building block employs Mobile Inverted Bottleneck Convolution (MBConv) with expansion ratio *t*, kernel size *k*, and stride *s*:

Expansion Phase:

(3) $$\begin{array}{cc} F_{exp} & ={\mathrm{Conv}}_{1\times 1}\left(F_{in}\right)\cdot t, \end{array}$$

(4) $$\begin{array}{cc} F_{exp} & =\mathrm{BatchNorm}(\mathrm{Swish}\left(F_{exp}\right)); \end{array}$$

Depthwise Convolution:

(5) $$\begin{array}{cc} F_{dw} & ={\mathrm{DepthwiseConv}}_{k\times k}\left(F_{exp},\mathrm{stride}=s\right), \end{array}$$

(6) $$\begin{array}{cc} F_{dw} & =\mathrm{BatchNorm}(\mathrm{Swish}\left(F_{dw}\right)); \end{array}$$

Squeeze-and-Excitation Attention:

(7) $$\begin{array}{cc} F_{se} & =\mathrm{SE}\left(F_{dw}\right)=\sigma \left({\mathrm{FC}}_{2}\left(\mathrm{ReLU}\left({\mathrm{FC}}_{1}\left(\mathrm{GAP}\left(F_{dw}\right)\right)\right)\right)\right), \end{array}$$

(8) $$\begin{array}{cc} F_{att} & =F_{dw}⊙F_{se}; \end{array}$$

Projection Phase:

(9) $$\begin{array}{cc} F_{proj} & ={\mathrm{Conv}}_{1\times 1}\left(F_{att}\right), \end{array}$$

(10) $$\begin{array}{cc} F_{out} & =F_{proj}+F_{in}\;(\mathrm{if}\;\mathrm{stride}=1\;\mathrm{and}\;\mathrm{input}/\mathrm{output}\;\mathrm{dimensions}\;\mathrm{match}), \end{array}$$

where GAP denotes global average pooling, σ is the sigmoid function, ${\mathrm{FC}}_{1}$ and ${\mathrm{FC}}_{2}$ are fully connected layers with reduction ratio r=4, and ⊙ represents element-wise multiplication.

The Squeeze-and-Excitation mechanism adaptively recalibrates channel-wise feature responses by explicitly modeling interdependencies between channels:

(11) $$\mathrm{SE}\left(F\right)=F⊙\sigma (W_{2}\delta \left(W_{1}z\right)),$$

where $z=\frac{1}{H\times W}\sum_{i=1}^{H}\sum_{j=1}^{W}F\left(i,j\right)$ is the global average pooled feature, $W_{1}\in R^{\frac{C}{r}\times C}$ and $W_{2}\in R^{C\times \frac{C}{r}}$ are learned parameters, and δ denotes the ReLU activation.

Multi-Scale Feature Extraction: EfficientNet generates hierarchical feature representations at multiple scales through progressive downsampling:

(12) $$\begin{array}{cc} C_{2} & ={\mathrm{MBConv}}_{1,3,1}\left(F_{input}\right)\;(\mathrm{stride}=1,\;\mathrm{output}:\;\frac{H}{4}\times \frac{W}{4}), \end{array}$$

(13) $$\begin{array}{cc} C_{3} & ={\mathrm{MBConv}}_{6,3,2}\left(C_{2}\right)\;(\mathrm{stride}=2,\;\mathrm{output}:\;\frac{H}{8}\times \frac{W}{8}), \end{array}$$

(14) $$\begin{array}{cc} C_{4} & ={\mathrm{MBConv}}_{6,5,2}\left(C_{3}\right)\;(\mathrm{stride}=2,\;\mathrm{output}:\;\frac{H}{16}\times \frac{W}{16}), \end{array}$$

(15) $$\begin{array}{cc} C_{5} & ={\mathrm{MBConv}}_{6,3,2}\left(C_{4}\right)\;(\mathrm{stride}=2,\;\mathrm{output}:\;\frac{H}{32}\times \frac{W}{32}), \end{array}$$

where the subscripts indicate expansion ratio, kernel size, and stride, respectively.

Model Variants and Channel Dimensions: We explore three EfficientNet variants (B3, B5, B7) with varying computational complexity and feature representation capacity. Table 2 presents the channel dimensions at stages 2, 3, 4, and 5 for each variant. The backbone supports flexible multi-scale feature extraction from any combination of these stages. In our final architecture configuration, we extract features from stages {3,4,5} using out_indices = (3, 4, 5), while ablation studies in Section 5.2 explore alternative stage combinations including out_indices = (2, 3, 4, 5).

This architecture achieves superior parameter efficiency with EfficientNet-B3 requiring only 10.1 M parameters while delivering competitive accuracy, representing an 2.33× parameter reduction compared to ResNet-50.

### 3.3. Neck

We employ a lightweight ChannelMapper neck architecture to efficiently bridge the backbone and detection head by unifying multi-scale feature representations into a consistent channel dimension. Unlike complex feature pyramid networks (FPNs) [42] that require extensive lateral connections and top–down pathways, our ChannelMapper provides a streamlined alternative that achieves comparable performance with significantly reduced computational overhead.

The ChannelMapper neck is designed to achieve efficient multi-scale feature fusion through direct channel-wise transformation while maintaining spatial information integrity. The neck consists of three key components that work synergistically to provide unified feature representations: (1) adaptive channel normalization for harmonizing heterogeneous feature dimensions from the backbone, (2) group normalization with learnable affine parameters for stable feature statistics, and (3) progressive pyramid construction for generating additional feature scales through controlled downsampling.

Given the hierarchical feature maps $\left\{C_{3},C_{4},C_{5}\right\}$ extracted from EfficientNet backbone at stages 3, 4, and 5 with respective channel dimensions $\left\{C_{3},C_{4},C_{5}\right\}$ that vary by model variant (see Table 2 in Section 3.2): {48,136,384} for EfficientNet-B3, {64,176,512} for EfficientNet-B5, and {80,224,640} for EfficientNet-B7, the ChannelMapper performs efficient channel-wise transformation to a unified dimension $C_{out}=256$.

Channel Normalization: For each feature level i∈{3,4,5}, we apply 1×1 convolution to project features to a unified dimension:

(16) $$F_{i}^{norm}={\mathrm{Conv}}_{1\times 1}\left(C_{i};W_{i}\in R^{C_{out}\times C_{i}}\right),$$

where $C_{i}$ represents the variant-specific input channel dimension and $C_{out}=256$ is the target unified channel dimension across all variants.

Group Normalization: To ensure training stability and feature consistency across scales, we apply Group Normalization with 32 groups:

(17) $$F_{i}^{GN}=\mathrm{GN}\left(F_{i}^{norm}\right)=\gamma \frac{F_{i}^{norm}-\mu_{g}}{\sqrt{\sigma_{g}^{2}+\epsilon }}+\beta ,$$

where $\mu_{g}$ and $\sigma_{g}^{2}$ are the mean and variance computed within each group *g*, γ and β are learnable affine parameters, and $\epsilon =10^{-5}$ is a small constant for numerical stability. The feature channels are divided into G=32 groups, with each group containing $\frac{256}{32}=8$ channels.

Multi-Scale Pyramid Construction: The ChannelMapper generates a 4-level feature pyramid $\left\{P_{3},P_{4},P_{5},P_{6}\right\}$ where the first three levels are directly mapped from backbone outputs:

(18) $$\begin{array}{cc} P_{3} & =F_{3}^{GN}\in R^{\frac{H}{8}\times \frac{W}{8}\times 256}, \end{array}$$

(19) $$\begin{array}{cc} P_{4} & =F_{4}^{GN}\in R^{\frac{H}{16}\times \frac{W}{16}\times 256}, \end{array}$$

(20) $$\begin{array}{cc} P_{5} & =F_{5}^{GN}\in R^{\frac{H}{32}\times \frac{W}{32}\times 256}, \end{array}$$

and the fourth level is constructed through stride-2 convolution with preserved channel dimension:

(21) $$P_{6}=\mathrm{GN}\left({\mathrm{Conv}}_{3\times 3}\left(P_{5},\mathrm{stride}=2,\mathrm{padding}=1\right)\right)\in R^{\frac{H}{64}\times \frac{W}{64}\times 256}.$$

Note that the kernel size for additional pyramid levels is fixed at 3×3 in the ChannelMapper implementation, regardless of the kernel size parameter used for initial channel mapping.

Computational Efficiency Analysis: The ChannelMapper architecture offers substantial computational advantages over traditional FPN-based necks. The parameter count for channel mapping operations is

(22) $$N_{params}=\sum_{i\in \{3,4,5\}}\left(C_{i}\times C_{out}\times 1\times 1\right)=\left(80+224+640\right)\times 256=241,664,$$

representing a significant reduction compared to FPN architectures that require lateral connections, top–down pathways, and additional convolution layers. The computational complexity (FLOP) for feature level *i* with spatial dimensions $H_{i}\times W_{i}$ is

(23) $${\mathrm{FLOPs}}_{i}=C_{i}\times C_{out}\times H_{i}\times W_{i}.$$

Feature Representation Quality: Despite its architectural simplicity, the ChannelMapper preserves rich semantic information through direct feature transformation without lossy fusion operations. The unified 256-dimensional representation provides the following:

- Consistent feature statistics across scales through group normalization, facilitating stable gradient flow during training;
- Preserved spatial resolution at each pyramid level without interpolation artifacts;
- Direct gradient pathways from detection head to backbone, enabling efficient end-to-end optimization.

This lightweight neck architecture achieves a 3.8× parameter reduction compared to standard FPN while maintaining competitive feature representation quality, making it particularly suitable for deployment scenarios requiring minimal computational overhead and maximum inference efficiency.

### 3.4. DELTA Head

**Figure 2:**
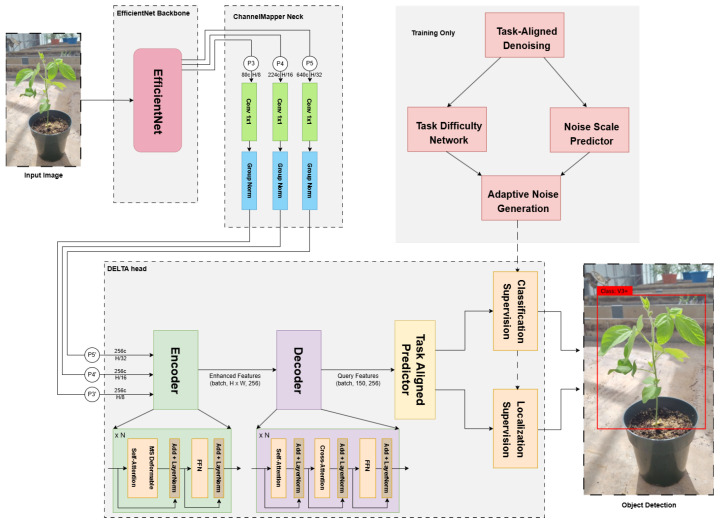
Overall architecture of the proposed DELTA-SoyStage detection framework. The system consists of four main components: (1) EfficientNet backbone for feature extraction, (2) ChannelMapper neck for multi-scale feature aggregation, (3) DELTA head with encoder–decoder architecture and task-aligned predictors, and (4) task-aligned denoising module (training only) for robust learning through adaptive noise generation.

We propose the DELTA (Denoising Enhanced Lightweight Task Alignment) head, a novel detection head that synergistically combines the task decomposition mechanism from TOOD [46] with the denoising training strategy from DINO [45]. Figure 2 illustrates the overall architecture including our proposed DELTA head.

The DELTA head is designed to achieve superior detection performance while maintaining computational efficiency. Operating on multi-scale features from the ChannelMapper neck, the DELTA head consists of six key components: (1) a multi-scale deformable transformer encoder for feature aggregation, (2) a query-based decoder with iterative refinement, (3) task-aligned predictors for classification and localization, (4) classification supervision with Quality Focal Loss, (5) localization supervision with geometric constraints, and (6) task-aligned denoising with adaptive noise generation for training robustness.

Encoder: Given multi-scale features $F=\{F_{3},F_{4},F_{5}\}$ with 256 channels from the neck, each encoder block applies multi-scale deformable self-attention followed by a feed-forward network, both with residual connections:

$$H_{l}^{\mathrm{attn}}=\mathrm{LN}\left(\mathrm{MSDeformAttn}\left(H_{l-1}\right)+H_{l-1}\right),$$

$$H_{l}=\mathrm{LN}\left(\mathrm{FFN}\left(H_{l}^{\mathrm{attn}}\right)+H_{l}^{\mathrm{attn}}\right),$$

where MSDeformAttn aggregates information across three feature levels, and LN denotes layer normalization. We present three DELTA-SoyStage (DSS) variants with different encoder–decoder configurations: DSS-S uses 2 encoder blocks, while DSS-M and DSS-L use 3 encoder blocks.

Decoder: The decoder refines object queries Q through *N* blocks, each consisting of self-attention, cross-attention to encoded features, and a feed-forward network:

$$Q_{l}^{\mathrm{self}}=\mathrm{LN}\left(\mathrm{SelfAttn}\left(Q_{l-1}\right)+Q_{l-1}\right),$$

$$Q_{l}^{\mathrm{cross}}=\mathrm{LN}\left(\mathrm{CrossAttn}\left(Q_{l}^{\mathrm{self}},H,R_{l}\right)+Q_{l}^{\mathrm{self}}\right),$$

$$Q_{l}=\mathrm{LN}\left(\mathrm{FFN}\left(Q_{l}^{\mathrm{cross}}\right)+Q_{l}^{\mathrm{cross}}\right),$$

where $R_{l}$ are iteratively updated reference points. DSS-S and DSS-M use a single decoder block (1), while DSS-L employs three decoder blocks (3) for enhanced refinement capacity.

Task-Aligned Predictors: The predictors leverage distinct feature representations optimized for classification and localization tasks.

Classification Predictor:

$$F_{\mathrm{cls}}=Q_{l}+0.1\cdot \tanh\left(Q_{l}\right),$$

$$P_{\mathrm{cls}}={\mathrm{MLP}}_{\mathrm{cls}}\left(F_{\mathrm{cls}}\right);$$

Localization Predictor:

$$F_{\mathrm{reg}}=Q_{l}+0.1\cdot \sigma \left(Q_{l}\right),$$

$$P_{\mathrm{bbox}}={\mathrm{MLP}}_{\mathrm{reg}}\left(F_{\mathrm{reg}}\right),$$

where ${\mathrm{MLP}}_{\mathrm{cls}}$ and ${\mathrm{MLP}}_{\mathrm{reg}}$ are compact multi-layer perceptrons with 128 hidden dimensions (reduced from 256 in standard detection heads for parameter efficiency).

Classification Supervision: We employ Quality Focal Loss (QFL) to jointly optimize classification score and localization quality:

$$L_{\mathrm{cls}}=-{\left|y-\sigma \right|}^{\beta }\left(\left(1-\sigma \right)\log\left(1-\sigma \right)+\sigma \log\left(\sigma \right)\right),$$

where *y* is the IoU-aware quality label, σ is the predicted classification score, and β=2.0 controls the modulation factor.

Localization Supervision: The localization loss combines L1 and Generalized IoU (GIoU) losses:

$$L_{\mathrm{reg}}=\lambda_{l1}L_{L1}\left(b,\hat{b}\right)+\lambda_{\mathrm{giou}}L_{\mathrm{GIoU}}\left(b,\hat{b}\right),$$

where $\lambda_{l1}=5.0$, $\lambda_{\mathrm{giou}}=2.0$, and b, $\hat{b}$ are ground truth and predicted bounding boxes in (x,y,w,h) format, respectively.

Task-aligned Adaptive Denoising: To address the class imbalance shown in Table 1, we employ an adaptive denoising strategy that scales both label and bounding box perturbations inversely with class frequency. The class-specific scaling factor is computed as

$$\alpha_{c}=1.5-\frac{n_{c}}{n_{\max}},$$

where $n_{c}$ is the frequency of class *c* and $n_{\max}$ is the frequency of the most common class. Label perturbations respect temporal ordering by limiting shifts to adjacent stages:

$$\Delta ∼U_{\mathrm{discrete}}\left(-\left\lfloor 2\alpha_{c}\right\rfloor ,\left\lfloor 2\alpha_{c}\right\rfloor \right),$$

while bounding boxes are perturbed as

$$\tilde{b}=b_{\mathrm{gt}}+\alpha_{c}\cdot \eta \cdot u,$$

where $b_{\mathrm{gt}}$ is the ground truth bounding box, $u∼U{\left(-1,1\right)}^{4}$ is a uniform random vector, and η=1.0 is the noise scale parameter. This adaptive strategy ensures that rare growth stages receive stronger perturbations to improve their representation during training, while abundant stages avoid over-perturbation, thereby balancing training attention across classes while maintaining biological plausibility of the augmented samples.

This architecture achieves a 50% reduction in parameters while maintaining competitive performance through task decomposition and intelligent adaptive denoising strategies.

## 4. Experiments

### 4.1. Implementation Details

All experiments are implemented using the PyTorch deep learning framework [48] in conjunction with MMDetection [49], an open-source toolbox for object detection. Training was conducted on a high-performance computing cluster equipped with NVIDIA A100 80 GB PCIe GPU, an Intel Xeon Gold 6240 CPU at 2.60 GHz and 32 GB of Memory. The software environment consists of Python 3.8.20, PyTorch 2.4.1 (CUDA 12.4, cuDNN 9.1.0), TorchVision 0.20.0, MMEngine 0.10.7, and OpenCV 4.11.0. Compilation is performed using GCC 11.5.0 with Intel MKL and AVX512 support. All models are trained and evaluated using a single GPU per job. To ensure reproducibility, we fix the random seed to 115045267 and use identical data splits across all experiments.

We benchmark the 9 model-backbone combinations introduced in Section 3—comprising three detection models (DINO, TOOD, and Faster R-CNN) paired with three backbones (ResNet-50, ResNet-101, and Swin Transformer)—alongside our proposed DELTA-SoyStage model that combines EfficientNet backbone with the DELTA head.

All models utilize pretrained backbones initialized from ImageNet. For the 9 baseline combinations, we employ a base learning rate of $1\times 10^{-4}$ with the AdamW optimizer, following the hyperparameter recommendations from the DINO detector [45]. Given the significantly smaller parameter count of our proposed DELTA-SoyStage (due to the compact EfficientNet backbone and 50% parameter reduction in the DELTA head), we use a higher base learning rate of $2\times 10^{-4}$ to ensure effective optimization of the lightweight architecture, a common practice when training smaller models [47].

Each model is trained for 50 epochs with a weight decay of $1\times 10^{-4}$, consistent with standard detection training protocols [45]. A linear warmup phase over the first 1000 iterations (starting from 0.1% of the base learning rate) is followed by a multi-step learning rate decay at epochs 30 and 40 (decay factor 0.1), a standard schedule for moderate-length training regimes. A parameter-wise learning rate multiplier of 0.1 is applied to all backbones to preserve pretrained features. This differentiated learning rate strategy accounts for the substantial architectural differences while maintaining fair comparative evaluation across all model configurations.

To stabilize training, gradient clipping is applied with a maximum norm of 0.1 (L2 norm). All models are trained with a batch size of 4 images per GPU. Input images are resized to 512 × 512 pixels while preserving aspect ratio. Data augmentation is performed using the Albumentations library [50], including random horizontal flipping, scaling, and photometric distortions (brightness, contrast, saturation, hue) to simulate the variability of greenhouse imaging conditions.

For loss functions, we use Quality Focal Loss [51] for classification and GIoU Loss [52] for bounding box regression. Validation is performed every 10 epochs, and checkpoints are saved based on the best mAP on the validation set using the COCO evaluation protocol.

### 4.2. Evaluation Metrics

To ensure comprehensive and fair comparison across all model configurations, we evaluate detection performance using the COCO evaluation protocol [53], which is widely adopted for object detection benchmarks and provides robust assessment across multiple IoU thresholds and object scales. Our evaluation encompasses both accuracy and efficiency metrics to provide a holistic view of model performance suitable for agricultural deployment scenarios.

Detection Performance Metrics: We report six primary detection metrics following the COCO evaluation standard, each designed to assess different aspects of detection quality. Table 3 provides detailed descriptions of all evaluation metrics used in our experiments.

The primary metric AP (Average Precision) is computed as the area under the precision-recall curve averaged across IoU thresholds from 0.50 to 0.95 with step size 0.05:

(24) $$\mathrm{AP}=\frac{1}{10}\sum_{t=0.50}^{0.95}{\mathrm{AP}}^{\left(t\right)},$$

where ${\mathrm{AP}}^{\left(t\right)}$ represents the average precision at IoU threshold *t*. This metric provides a comprehensive assessment of both localization accuracy and classification performance.

Scale-Specific Evaluation: The scale-specific metrics (AP_*S*_, AP_*M*_, AP_*L*_) are particularly relevant for agricultural applications where soybean plants exhibit significant size variations across growth stages. Object scale classification is based on bounding box area *A*:

(25) $$\begin{array}{cc} \mathrm{Small}\;\mathrm{objects}:\; & A<32^{2}=1024\;{\mathrm{pixels}}^{2} \end{array}$$

(26) $$\begin{array}{cc} \mathrm{Medium}\;\mathrm{objects}:\; & 32^{2}\leq A<96^{2}=9216\;{\mathrm{pixels}}^{2} \end{array}$$

(27) $$\begin{array}{cc} \mathrm{Large}\;\mathrm{objects}:\; & A\geq 96^{2}\;{\mathrm{pixels}}^{2} \end{array}$$

These thresholds enable detailed analysis of model performance across different plant sizes, from early vegetative stages (typically small) to mature reproductive stages (typically large).

Computational Efficiency Assessment: Beyond accuracy metrics, we evaluate computational efficiency through three key measures essential for practical deployment. All reported efficiency metrics refer to inference-time requirements.

Model Parameters: The total number of trainable parameters provides insight into model complexity and memory requirements:

(28) $$\mathrm{Parameters}=\sum_{l=1}^{L}|W_{l}\left|+\right|b_{l}|,$$

where $W_{l}$ and $b_{l}$ represent the weight matrices and bias vectors for layer *l*, and *L* is the total number of layers.

Floating Point Operations (FLOPs): We measure computational cost through FLOPs required for single forward inference, calculated as

(29) $$\mathrm{FLOPs}=\sum_{l=1}^{L}{\mathrm{FLOPs}}_{l}=\sum_{l=1}^{L}\left({\mathrm{MAC}}_{l}\times {\mathrm{Output\_Size}}_{l}\right),$$

where ${\mathrm{MAC}}_{l}$ represents multiply-accumulate operations for layer *l*.

Reporting Protocol: All metrics are computed on the test set with consistent evaluation conditions: input resolution of 512×512 pixels, confidence threshold of 0.05, and NMS threshold of 0.5. This comprehensive evaluation framework enables fair comparison across different architectural choices while providing insights relevant to real-world agricultural deployment scenarios.

Computational Efficiency Score: To provide a unified assessment of the accuracy to computational cost trade-off, we introduce a comprehensive efficiency score that considers both model complexity and computational requirements. This metric enables direct comparison across architectures with vastly different parameter counts and FLOP requirements, which is essential for agricultural edge deployment where resource constraints vary significantly. The efficiency score is calculated as

(30) $$\mathrm{Efficiency}\;\mathrm{Score}=\frac{\mathrm{AP}}{(w_{p}\times \frac{\mathrm{Params}}{100M}+w_{f}\times \frac{\mathrm{FLOPs}}{10G})\times \mathrm{Normalization}\;\mathrm{Factor}}$$

where $w_{p}$ and $w_{f}$ represent the weighting factors for parameters and FLOPs respectively, with $w_{p}+w_{f}=1$. Based on resource scarcity analysis for agricultural edge deployment, we set $w_{p}=0.2$ and $w_{f}=0.8$, reflecting that computational efficiency is the primary constraint rather than memory capacity in agricultural edge scenarios. Typical edge devices provide abundant memory for object detection models (0.1–0.5 GB requirements), while sustained computational performance faces higher utilization pressure from continuous inference workloads. The normalization factor scales all scores relative to a baseline model, enabling meaningful comparison across different architectural approaches. The denominators (100 M for parameters, 10 G for FLOPs) provide scaling to ensure balanced contributions from both components. Higher efficiency scores indicate superior accuracy-to-cost ratios, with models achieving better detection performance while minimizing computational overhead essential for sustainable field operation and extended battery life in autonomous agricultural systems.

## 5. Results

### 5.1. Quantitative Results

Table 4 presents a comprehensive performance comparison across baseline detection methods and our proposed DELTA-SoyStage variants, encompassing multiple evaluation metrics for 12 model-backbone combinations. The baseline results demonstrate significant variations in both accuracy and computational efficiency across different architectural choices. DINO with Swin Transformer achieves strong performance with an AP of 74.7%, followed closely by DINO with ResNet-101 (74.4%). Among single-stage detectors, TOOD with ResNet-101 shows competitive performance (72.5% AP), while TOOD with Swin Transformer achieves 68.1% AP. Faster R-CNN, despite being a two-stage detector, shows moderate performance with the best result of 69.9% AP using the Swin Transformer backbone.

Regarding computational efficiency among baseline methods, the results reveal a clear trade-off between accuracy and model size. ResNet-based models maintain relatively compact parameter counts (32–60 M parameters), while Swin Transformer configurations require slightly more parameters (56–66 M) with substantially higher computational costs. The FLOP counts reflect this efficiency-accuracy trade-off, with lighter models requiring 28–52 GFLOPs compared to 44–102 GFLOPs for Swin Transformer configurations.

Our proposed DELTA-SoyStage variants, shown in the lower section of Table 4, demonstrate exceptional computational efficiency while achieving highly competitive detection accuracy. DELTA-SoyStage with EfficientNet-B7 achieves 73.9% AP using only 24.4 GFLOPs, representing a 14% reduction in computational cost compared to the most efficient baseline (TOOD-ResNet-50: 28.5 GFLOPs) while delivering substantially superior accuracy (73.9% vs. 66.2%). More significantly, when compared to the best-performing baseline, DSS-B7 (DELTA-SoyStage B7) requires 4.2× fewer FLOPs than DINO-Swin (102.5 GFLOPs) for only 0.8% accuracy difference (74.7% vs. 73.9%), demonstrating that our architecture achieves near-state-of-the-art accuracy with dramatically reduced computational overhead.

The efficiency advantages extend across all model variants as demonstrated quantitatively in Table 5. Our comprehensive efficiency analysis reveals that DSS-L achieves an exceptional 2.79× efficiency score compared to the high-accuracy baseline (DINO-Swin), while maintaining highly competitive accuracy within 0.8% of the best-performing model. When compared to methods of similar accuracy, DSS-L demonstrates 1.04× better efficiency than DINO-ResNet-50 and 1.27× better efficiency than TOOD-ResNet-101, validating our approach’s superior computational optimization. DSS-M achieves 70.3% AP with merely 15.2 GFLOPs, demonstrating an outstanding 4.76× efficiency score that outperforms all baseline methods while requiring 1.9× fewer FLOPs than the most efficient ResNet-based approaches. Most remarkably, our lightest variant DSS-S operates at an exceptional 8.7 GFLOPs while maintaining 65.7% AP, achieving an unprecedented 8.44× efficiency score that establishes a new efficiency frontier for agricultural detection applications.

The efficiency gains stem from our strategic focus on FLOP optimization rather than parameters reduction as evidenced by the 20/80 parameter/FLOP weighting used in our efficiency calculation. While DSS-L requires 70.5 M parameters compared to 41.5 M for DINO-ResNet-50, the dramatic FLOP reduction (24.4 G vs. 32.6 G) dominates the efficiency calculation, resulting in superior overall performance for edge deployment scenarios. This validates our architectural design choices, particularly the lightweight ChannelMapper neck and efficient encoder–decoder scaling in the DELTA head. The combination of EfficientNet’s compound scaling with our streamlined detection framework enables deployment on resource-constrained agricultural platforms without compromising detection quality essential for precision farming applications.

### 5.2. Ablation Study

To validate our architectural design choices and understand the contribution of individual components to overall performance, we conduct comprehensive ablation studies on our DELTA-SoyStage model variants. These experiments systematically isolate and evaluate specific design decisions to provide insights into the optimal configuration for agricultural edge deployment. Our ablation analysis focuses on three critical aspects of the DELTA-SoyStage architecture: the effectiveness of task alignment and adaptive noise mechanisms in the detection head, the impact of multi-scale feature extraction depth from the EfficientNet backbone, and the optimal encoder–decoder architecture scaling for transformer-based processing. Each study is conducted across all three DELTA-SoyStage variants (Small, Medium, Large) to ensure findings generalize across different model complexities and computational budgets. These ablations provide empirical justification for our design choices while identifying opportunities for further optimization in resource-constrained agricultural scenarios.

Task Alignment and Adaptive Noise Components: We ablate two DELTA head components—(i) task alignment, which reduces the classification–localization gap via task-specific feature decomposition, and (ii) adaptive noise, which modulates training noise to improve robustness. We systematically evaluate four configurations: both components enabled (True, True), task alignment only (True, False), adaptive noise only (False, True), and both disabled (False, False). This isolates their individual and joint effects, showing whether they are complementary or if one dominates, and clarifies the accuracy–complexity trade-off for resource-constrained agricultural deployments.

The task alignment and adaptive noise ablation reveals model size-dependent optimization patterns that provide important insights into component effectiveness across different architectural complexities. For the smallest variant (DSS-S), counterintuitively, disabling both components yields the best performance (65.9% AP) as shown in Table 6, suggesting that the limited model capacity benefits from simplified training dynamics rather than sophisticated alignment mechanisms. This indicates potential overfitting or optimization difficulties when complex training strategies are applied to lightweight architectures.

The medium variant (DSS-M) demonstrates the most robust behavior, with both the full configuration (task alignment + adaptive noise) and adaptive noise alone achieving optimal 69.5% AP. This suggests that DSS-M represents a sweet spot where the model has sufficient capacity to benefit from training enhancements without suffering from optimization complexity. The consistent performance across configurations indicates stable training dynamics at this scale.

For the largest variant (DSS-L), the full configuration with both components enabled clearly outperforms all alternatives (72.3% AP vs. 70.8–71.9% for partial configurations), demonstrating that high-capacity models can effectively leverage sophisticated training mechanisms. The substantial performance degradation when both components are disabled (69.5% AP, a 2.8 percentage point drop) confirms that large models require advanced training strategies to reach their full potential.

These findings suggest an optimal component selection strategy based on deployment requirements: lightweight models (B3) benefit from simplified training, medium models (B5) show flexibility across configurations, and large models (B7) require full training enhancement for optimal performance. This has practical implications for agricultural deployment where different computational budgets may favor different DELTA-SoyStage variants with correspondingly adjusted training strategies.

Multi-Scale Feature Extraction Depth: This ablation examines the impact of incorporating additional shallow features from the EfficientNet backbone by expanding from three feature levels (stages 3, 4, 5) to four levels (stages 2, 3, 4, 5). The inclusion of stage 2 features provides finer spatial resolution (H/8 × W/8) that potentially benefits detection of small soybean plants in early growth stages, though at the cost of increased computational overhead and memory usage. Shallow features typically contain more spatial detail but less semantic information, making this trade-off particularly relevant for agricultural applications where plant size varies dramatically across growth stages. The four-level configuration increases the number of feature pyramid levels in the ChannelMapper neck, requiring additional channel normalization and processing. This study determines whether the enhanced spatial detail from stage 2 features justifies the computational cost increase, providing insights into the optimal balance between detection sensitivity for small objects and overall system efficiency.

The multi-scale feature extraction depth ablation definitively validates our design choice of using three feature levels (stages 3, 4, 5) over four levels (stages 2, 3, 4, 5) from the EfficientNet backbone. Across all DELTA-SoyStage variants, the inclusion of stage 2 features consistently degrades detection performance, with particularly severe impact on smaller models. DSS-S experiences the most dramatic performance loss (11.3 percentage points: 65.7% → 54.4% AP) as shown in Table 7, indicating that lightweight architectures are especially sensitive to noisy shallow features that lack sufficient semantic content for effective object detection. The medium variant DSS-M shows moderate degradation (5.7 percentage points: 69.5% → 63.8% AP), while the large variant DSS-L demonstrates relative resilience with minimal performance loss (1.5 percentage points: 72.3% → 70.8% AP), suggesting that higher-capacity models can better handle suboptimal feature inputs but still benefit from the streamlined approach. The consistent performance degradation across all scale-specific metrics (AP_*S*_, AP_*M*_, AP_*L*_) confirms that stage 2 features provide insufficient semantic information to justify their computational overhead and potential optimization difficulties. These results align with established computer vision principles that shallow features, while containing fine spatial detail, often introduce noise and computational burden without corresponding accuracy benefits in object detection tasks. This ablation empirically justifies our architectural decision to exclude stage 2 features, supporting our efficiency-focused design philosophy while maintaining optimal detection accuracy for agricultural applications.

Encoder–Decoder Architecture Scaling: This ablation investigates the optimal depth configuration for the transformer-based encoder and decoder components within our DELTA head. The encoder processes multi-scale features through self-attention mechanisms to capture global dependencies and enhance feature representations, while the decoder generates final detection predictions through cross-attention between learned queries and encoded features. We systematically vary the number of encoder and decoder layers to understand the trade-off between model capacity and computational efficiency. Deeper architectures typically provide enhanced representational power and improved handling of complex spatial relationships, but at the cost of increased parameters, computational load, and potential overfitting risk. For agricultural edge deployment, this balance is particularly critical as the model must achieve sufficient accuracy to distinguish subtle morphological differences between growth stages while maintaining real-time inference capabilities on resource-constrained hardware. This analysis identifies the minimal architecture depth required to achieve optimal performance, informing deployment decisions for various computational budget scenarios.

The encoder–decoder architecture depth ablation reveals distinct optimal configurations across DELTA-SoyStage variants, with our final selections prioritizing maximum detection accuracy essential for agricultural applications (Table 8). For the small variant, DSS-S with 2 encoders and 1 decoder achieves optimal performance with 65.7% AP and the highest efficiency score of 8.45×, demonstrating that lightweight models benefit from streamlined architectures that avoid optimization difficulties associated with deeper transformer layers. The medium variant (DSS-M) shows nuanced behavior where DSS-M with 3 encoders and 1 decoder achieves the highest accuracy (70.3% AP) compared to the 2 encoder, 1 decoder configuration (69.5% AP), and we select this configuration for our final model despite slightly lower efficiency (4.76× vs. 5.29×), as the 0.8% accuracy improvement is significant for agricultural detection tasks. For the large variant, DSS-L with 3 encoders and 3 decoders delivers peak accuracy (73.9% AP), substantially outperforming the minimal 2-encoder, 1-decoder setup (72.3% AP), and we adopt this as our final configuration prioritizing the 1.6% accuracy gain over the efficiency difference (2.79× vs. 3.01×). Notably, 4-encoder configurations consistently underperform across all variants, with DSS-L with 4 encoders and 1 decoder showing catastrophic accuracy degradation (56.2% AP), indicating that excessive encoder depth leads to optimization instability.

Final Architectural Configuration and Deployment Recommendations Based on our comprehensive ablation studies, we establish variant-specific optimal configurations that integrate encoder–decoder depth, training strategy, and feature extraction findings. Our accuracy-optimized selections are: DSS-S with 2 encoders, 1 decoder, simplified training (no task alignment/adaptive noise), and three-stage features (stages 3–5) achieving 65.7% AP; DSS-M with 3 encoders, 1 decoder, full training enhancement, and three-stage features achieving 70.3% AP; DSS-L with 3 encoders, 3 decoders, full training enhancement, and three-stage features achieving 73.9% AP.

For deployment scenarios prioritizing computational efficiency—such as edge devices, agricultural robots, or real-time monitoring systems with limited processing capabilities—we recommend alternative configurations. DSS-M can adopt 2 encoders and 1 decoder, achieving 69.5% AP with 5.29× efficiency (versus 70.3% AP and 4.76× efficiency), trading only 0.8% AP for 11.1% improved efficiency. DSS-L with 2 encoders and 1 decoder achieves 72.3% AP with 3.01× efficiency (versus 73.9% AP and 2.79× efficiency), sacrificing 1.6% AP for 7.9% efficiency gain. These efficiency-focused configurations maintain competitive performance while enabling deployment in resource-constrained environments where accuracy-optimized variants would be impractical. The choice between configurations depends on specific application requirements, with both thoroughly validated through our ablation studies.

### 5.3. Qualitative Analysis

**Figure 3:**
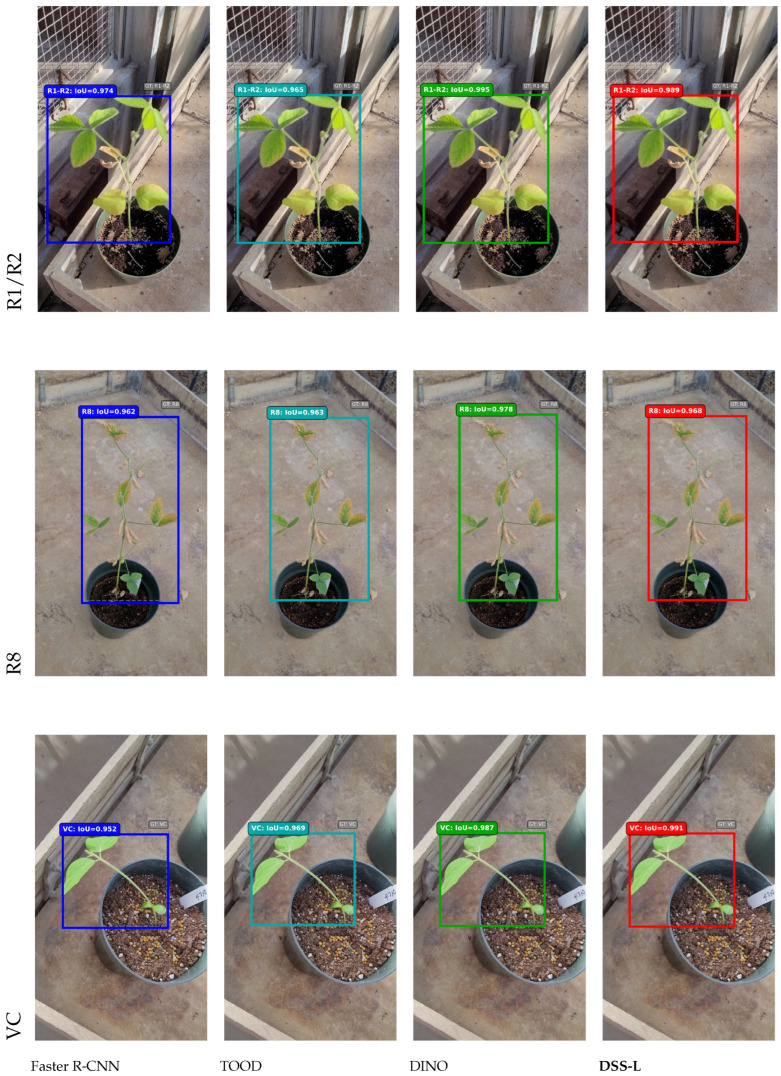
Qualitative comparison of detection results across soybean growth stages (R1–R2, R8, and VC) for state-of-the-art models. Each model uses distinct colored bounding boxes for predictions, while gray boxes indicate ground truth annotations. DSS-L achieves accurate detection with competitive IoU values compared to more complex models while maintaining superior computational efficiency.

Figure 3 presents qualitative detection results comparing DSS-L with state-of-the-art models across three representative soybean growth stages (R1–R2, R8, and VC). Faster R-CNN consistently displays perfect confidence scores (1.00) across all stages, which may indicate calibration issues despite high IoU values. TOOD demonstrates strong performance on early and late stages but shows notably reduced confidence on the R1–R2 flowering stage (0.829), suggesting difficulties in distinguishing subtle morphological features during reproductive transitions. DINO achieves the most competitive performance with IoU values ranging from 0.978 to 0.995, representing the strongest baseline for comparison.

DSS-L achieves highly competitive detection quality with IoU values ranging from 0.968 to 0.991 and well-calibrated confidence scores (0.907–0.945) across all growth stages. Notably, DSS-L attains the highest IoU (0.991) on the VC vegetative stage, outperforming all baselines including DINO (0.987). On the challenging R1–R2 flowering stage, DSS-L achieves 0.989 IoU with 0.907 confidence, demonstrating robust performance on reproductive stages where precise detection timing is critical for agricultural interventions. These results demonstrate the ability of DSS-L to maintain detection quality comparable to substantially larger models while offering superior computational efficiency, making it well-suited for deployment in resource-constrained precision agriculture applications.

## 6. Discussion

This work presents a novel DELTA (Denoising Enhanced Lightweight Task Alignment) detection head integrated with EfficientNet backbone for comprehensive soybean growth stage detection across nine phenological stages spanning the complete crop cycle. Our architecture achieves 73.9% AP with only 24.4 GFLOPs, demonstrating a 2.79× efficiency advantage over the high-accuracy baseline (DINO-Swin: 74.7% AP, 102.5 GFLOPs) with only 0.8% accuracy difference. The 50% parameter reduction in the DELTA head compared to standard DINO architectures, combined with strategic architectural optimizations including the lightweight ChannelMapper neck and efficient EfficientNet backbone, positions this model for deployment on resource-constrained agricultural edge devices. However, several limitations warrant acknowledgment: our dataset was collected exclusively in controlled greenhouse conditions, which may not fully capture field variability including weather fluctuations, diverse soil conditions, and pest pressure; additionally, class imbalance in the dataset, particularly for reproductive stages, may affect generalization performance. Moreover, the current study focuses on individual plant detection, which does not reflect real-world aerial survey scenarios involving dense canopies, overlapping plants, and occlusion common in precision agriculture deployments. This represents a fundamental limitation for practical UAV-based field monitoring applications. Future work will prioritize field validation using UAV-mounted RGB camera sensors to capture dense canopy scenarios with overlapping plants, occlusion handling, and aerial perspective imaging. Additional priorities include evaluation on larger public benchmark datasets, hardware optimization with inference speed measurements on specific edge computing platforms (NVIDIA Jetson, Raspberry Pi), and domain adaptation techniques to transfer from individual plant to multi-plant detection scenarios. Visual interpretability analysis using attention visualization techniques (e.g., Grad-CAM) to examine which morphological features the model prioritizes across different growth stages represents another important direction for enhancing model transparency and agronomic insights. Integration with agricultural IoT sensor ecosystems and the development of multi-modal sensor fusion architectures combining RGB camera sensor data with hyperspectral and environmental sensor data represent promising directions for advancing precision agriculture decision support systems. By enabling accurate, lightweight growth stage monitoring suitable for edge deployment, this work addresses a critical agricultural need by ensuring precise timing of herbicide and pesticide applications, preventing yield losses of 2.5–40% (USD 5–80/acre) from mistimed interventions while reducing environmental impact. This contributes to democratizing precision agriculture technologies for resource-limited farming operations.

## Figures and Tables

**Table 1:** Distribution of soybean growth stages in the dataset, showing both instance and image counts for each stage.

Growth Stage	Image Count	Description
VE	307	Emergence
VC	844	Cotyledon stage
V1	2597	First trifoliate leaf
V2	1035	Second trifoliate leaf
V3+	2909	Third trifoliate leaf
R1–R2	2195	Flowering stages
R3–R5	2220	Pod formation to seed fill
R6–R7	3810	Beginning maturity to physiological maturity
R8	1287	Full maturity
Total	17,204	-

**Table 2:** Channel dimensions for different EfficientNet variants at stages 2, 3, 4, and 5 with corresponding model complexity.

Variant	$C_{2}$	$C_{3}$	$C_{4}$	$C_{5}$	Parameters (M)
EfficientNet-B3 (Light)	32	48	136	384	10.1
EfficientNet-B5 (Medium)	40	64	176	512	27.29
EfficientNet-B7 (Large)	48	80	224	640	62.14

**Table 3:** Evaluation metrics used for comprehensive model assessment including detection accuracy and computational efficiency measures.

Metric	Description	Purpose
**Detection Accuracy Metrics**		
AP	Average Precision across IoU thresholds 0.50:0.95 with step 0.05	Overall detection quality
AP_50_	Average Precision at IoU threshold 0.50	Loose localization accuracy
AP_75_	Average Precision at IoU threshold 0.75	Precise localization accuracy
AP_*S*_	Average Precision for small objects (area $<32^{2}$ pixels)	Small-scale detection
AP_*M*_	Average Precision for medium objects ($32^{2}<$ area $<96^{2}$ pixels)	Medium-scale detection
AP_*L*_	Average Precision for large objects (area $>96^{2}$ pixels)	Large-scale detection
**Computational Efficiency Metrics**		
Parameters (M)	Total number of trainable parameters in millions	Model complexity
FLOPs (G)	Floating Point Operations in billions for single inference	Computational cost

**Table 4:** Comprehensive performance comparison of baseline detection models and our proposed DELTA-SoyStage variants across multiple backbone architectures. Results show accuracy metrics, detailed parameter breakdown, and computational requirements demonstrating the superior efficiency of DELTA.

Model	Backbone	Accuracy (%)					Parameters (M)			Efficiency
		AP ↑	${\mathrm{AP}}_{50}↑$	${\mathrm{AP}}_{75}↑$	${\mathrm{AP}}_{M}↑$	${\mathrm{AP}}_{L}↑$	B↓	N+H ↓	Total ↓	GFLOPs ↓
*Baseline Methods*										
DINO	ResNet-50	72.8	76.6	75.2	77.8	82.1	23.5	18.0	41.5	32.6
	ResNet-101	74.4	77.8	76.9	76.2	84.3	42.5	18.0	60.5	43.6
	Swin	**74.7**	**77.9**	**77.0**	**85.3**	83.8	48.8	14.7	63.5	102.5
Faster R-CNN	ResNet-50	65.6	74.8	73.0	49.6	77.6	23.5	17.9	41.4	41.7
	ResNet-101	69.1	75.7	73.7	57.4	81.1	42.5	17.9	60.4	52.6
	Swin	69.9	79.3	77.1	58.9	81.7	48.8	17.3	66.1	57.5
TOOD	ResNet-50	66.2	74.4	72.1	44.8	78.2	23.5	8.5	32.0	**28.5**
	ResNet-101	72.5	77.2	75.9	68.2	84.4	42.5	8.5	51.0	39.4
	Swin	68.1	76.0	73.6	55.9	79.9	48.8	8.0	56.8	44.7
*Our Proposed Method*										
DELTA-SoyStage	EfficientNet-B3	65.7	73.6	70.9	53.2	77.1	10.1	4.4	**14.5**	**8.7**
	EfficientNet-B5	70.3	76.7	74.5	59.9	82.2	27.3	5.5	**32.8**	**15.2**
	EfficientNet-B7	73.9	78.1	76.9	74.5	**85.1**	62.1	8.3	**70.5**	**24.4**

AP: Average Precision at IoU = 0.50:0.95; AP_50_: AP at IoU = 0.50; AP_75_: AP at IoU = 0.75; AP_*M*_: AP for medium objects; AP_*L*_: AP for large objects; B: Backbone parameters; N+H: Combined neck and head parameters; GFLOPs: Giga floating-point operations. Bold values indicate best performance in each category. DELTA-SoyStage variants achieve competitive accuracy with substantially reduced computational requirements. Arrows indicate optimization direction: ↑ denotes higher values are better; ↓ denotes lower values are better.

**Table 5:** Computational efficiency analysis comparing DELTA-SoyStage variants with state-of-the-art methods. Efficiency scores calculated with 20/80 parameter/FLOP weighting, normalized to DINO Swin baseline.

Method	AP ↑ (%)	Params ↓ (M)	GFLOPs ↓	FLOP ↑	Param ↑	Efficiency ↑
				Ratio	Ratio	Score
*Similar Accuracy Comparison (AP ≈ 72–74%)*						
DINO + ResNet-50	72.8	41.5	32.6	1.34×	0.59×	2.68×
TOOD + ResNet-101	72.5	51.0	39.4	1.62×	0.72×	2.20×
FRCNN + Swin	69.9	66.1	57.5	2.36×	0.94×	1.50×
**DSS-L**	**73.9**	**70.5**	**24.4**	**1.00×**	**1.00×**	**2.79×**
*Best Accuracy vs. Efficiency Trade-off*						
DINO + Swin	**74.7**	63.5	102.5	4.20×	0.90×	1.00×
**DSS-L**	73.9	**70.5**	**24.4**	**1.00×**	**1.00×**	**2.79×**
*Ultra-Efficient DELTA-SoyStage Variants*						
**DSS-M**	70.3	**32.8**	**15.2**	**0.62×**	**0.47×**	**4.76×**
**DSS-S**	65.7	**14.5**	**8.7**	**0.36×**	**0.21×**	**8.44×**
**DELTA-SoyStage Range vs. Best Baseline**	**65.7–73.9%**	**0.9–4.4× fewer**	**4.2–11.8× fewer**	**—**	**—**	**2.8–8.4× better**

Efficiency Score uses 20/80 parameter/FLOP weighting optimized for edge deployment. Normalized to DINO Swin baseline (1.00×). FLOP and parameter ratios relative to DSS-L. DELTA-SoyStage variants achieve 2.8–8.4× better efficiency through superior FLOP optimization, with DSS-S establishing unprecedented efficiency for agricultural applications. Bold values indicate best performance within each comparison category. Arrows indicate optimization direction: ↑ denotes higher values are better; ↓ denotes lower values are better.

**Table 6:** Task alignment and adaptive noise ablation study across DELTA-SoyStage variants. Results show the individual and combined impact of task alignment mechanisms and adaptive noise on detection performance.

Model	Task	Adaptive	AP ↑	${\mathrm{AP}}_{50}↑$	${\mathrm{AP}}_{75}↑$	${\mathrm{AP}}_{M}↑$	${\mathrm{AP}}_{L}↑$
Variant	Alignment	Noise					
DSS-S	✓	✓	65.7	73.6	70.9	53.2	77.1
	✓	×	65.3	73.9	69.9	55.3	76.0
	×	✓	64.2	73.0	69.0	47.6	75.4
	×	×	**65.9**	**74.0**	**70.7**	54.9	76.9
DSS-M	✓	✓	**69.5**	**77.8**	**74.3**	**58.2**	**81.2**
	✓	×	69.2	76.3	73.1	58.3	81.3
	×	✓	**69.5**	77.2	73.7	55.2	81.2
	×	×	68.3	76.3	73.0	57.9	79.9
DSS-L	✓	✓	**72.3**	**79.1**	**76.5**	**64.8**	**84.4**
	✓	×	70.8	77.9	75.3	65.6	83.0
	×	✓	71.9	78.3	75.8	69.0	83.4
	×	×	69.5	77.2	73.7	55.2	81.2

✓ indicates feature enabled; × indicates feature disabled. Bold values indicate best performance for each model variant across different component configurations. Arrows indicate optimization direction: ↑ denotes higher values are better.

**Table 7:** Multi-scale feature extraction depth ablation comparing 3-level vs. 4-level feature pyramid extraction from EfficientNet backbone across DELTA-SoyStage variants.

Model	Feature	AP ↑	${\mathrm{AP}}_{50}↑$	${\mathrm{AP}}_{75}↑$	${\mathrm{AP}}_{S}↑$	${\mathrm{AP}}_{M}↑$	${\mathrm{AP}}_{L}↑$
Variant	Layers	(%)	(%)	(%)	(%)	(%)	(%)
DSS-S	3	**65.7**	**73.6**	**70.9**	**0.9**	**53.2**	**77.1**
	4	54.4	61.2	57.5	0.3	21.2	65.7
DSS-M	3	**69.5**	**77.8**	**74.3**	**1.5**	**58.2**	**81.2**
	4	63.8	71.6	68.5	1.5	47.2	75.4
DSS-L	3	**72.3**	**79.1**	**76.5**	**2.9**	**64.8**	**84.4**
	4	70.8	78.8	74.8	2.0	63.6	82.8
**Performance**	**3 vs. 4**	**+5.7%**	**+6.6%**	**+6.7%**	**+0.5%**	**+10.9%**	**+5.4%**
**Difference**	**Average**						

Bold values indicate superior performance. Performance difference row shows average improvement of 3-layer over 4-layer configuration across all variants. Results demonstrate that additional shallow features degrade rather than enhance detection performance. Arrows indicate optimization direction: ↑ denotes higher values are better.

**Table 8:** Encoder–decoder architecture depth ablation study across DELTA-SoyStage variants. Results show the impact of varying encoder and decoder layer counts on detection performance and computational efficiency.

Model	Encoder	Decoder	AP ↑ (%)	Params ↓ (M)	GFLOPs ↓	Efficiency ↑
Variant	Layers	Layers				Score
*Small Variant (DSS-S)*						
DSS-S	2	1	**65.7**	**14.5**	**8.7**	**8.45**
	2	2	64.5	15.7	9.1	7.85
	3	1	48.0	15.3	11.1	5.10
	3	2	58.2	16.5	11.5	5.90
*Medium Variant (DSS-M)*						
DSS-M	2	1	69.5	**32.0**	**12.8**	**5.29**
	2	2	69.3	33.3	13.3	5.08
	3	1	**70.3**	32.8	15.2	4.76
	3	2	59.8	34.0	15.6	3.93
*Large Variant (DSS-L)*						
DSS-L	2	1	72.3	**67.2**	**21.2**	3.01
	2	2	73.4	68.5	21.6	3.00
	3	1	72.9	68.0	23.5	2.85
	3	2	72.5	69.2	24.0	2.78
	3	3	**73.9**	70.5	24.4	2.79
	4	1	56.2	68.7	25.9	2.07
	4	2	72.6	70.0	26.3	2.63
	4	3	68.5	71.2	26.7	2.44
	4	4	63.2	72.5	27.2	2.21

Efficiency scores calculated with 20/80 parameter/FLOP weighting, normalized to DINO Swin baseline. Bold values indicate optimal configurations for each variant considering both accuracy and efficiency trade-offs. Arrows indicate optimization direction: ↑ denotes higher values are better; ↓ denotes lower values are better.

## Data Availability

The data that support the findings of this study are available from the corresponding author upon reasonable request.
